# Cortical markers of excitation/inhibition balance are associated with sensory responsivity from infancy in longitudinal cohorts enriched for autism and ADHD

**DOI:** 10.1038/s41398-025-03791-9

**Published:** 2025-12-18

**Authors:** Virginia Carter Leno, Jannath Begum-Ali, Amy Goodwin, Luke Mason, Greg Pasco, Nisha Narvekar, Andrew Pickles, Tony Charman, Mark H. Johnson, Emily J. H. Jones, the STAARS Team, Mary Agyapong, Tessel Bazelmans, Leila Dafner, Mutluhan Ersoy, Laurel Fish, Teodora Gliga, Rianne Haartsen, Alexandra Hendry, Rebecca Holman, Sarah Kalwarowsky, Anna Kolesnik, Laura Pirazzoli, Chloë Taylor

**Affiliations:** 1 Institute of Psychiatry, Psychology & Neuroscience, King’s College London, London, UK; 2 Centre for Brain and Cognitive Development, Department of Psychological Sciences, Birkbeck, University of London, London, UK; 3 Department of Psychology, University of Cambridge, Cambridge, UK

**Keywords:** Predictive markers, Psychology

## Abstract

Autism and ADHD are characterised by atypical sensory responsivity, and this may be driven by alterations in the balance of cortical excitation to inhibition (E/I). Studies early in development are required to establish when sensory responsivity differences emerge and whether they predict later neurodevelopmental condition outcomes. We utilised data from a prospective longitudinal cohort of infants with and without a family history (FH) of autism and/or ADHD (N = 151; 55% male, 83% white). We extracted electroencephalography (EEG) metrics of E/I balance at 5, 10 and 14 months; the aperiodic exponent of the slope of the power spectrum (‘1/f’). Models estimated latent growth curves of parent-reported hyper and hypo-responsivity between 10 – 36 months. Analyses tested associations between developmental trajectories of FH, sensory responsivity, parent-rated neurodevelopmental traits at 3 years and E/I balance. We coded and entered binary variables indexing FH-autism and FH-ADHD in the same model, which allowed us to test for effects of one form of FH whilst adjusting for the impact of the other. Results showed that FH-autism was associated with greater increases in parent-reported hyper-responsivity between 10–36 months (over and above the effects of FH-ADHD), and in univariate models for hyper-responsivity only, the intercept and the slope of hyper-responsivity were positively associated with both autistic and ADHD traits at age 3 years. However, in joint models which included hypo-responsivity, associations between hyper-responsivity and autistic and ADHD traits became non-significant. In these joint models, FH-ADHD was associated with steeper increases in hypo-responsivity (over and above the effects of FH-autism). Higher hypo-responsivity at 10-month baseline was associated with both autistic and ADHD traits at 3 years. A steeper slope of hypo-responsivity predicted ADHD traits at 3 years. Males displayed higher baseline hypo-responsivity. Aperiodic exponent values at 5 and 10, but not 14 months, were associated with hyper-responsivity. Results suggest a dissociation in the type of sensory responsivity associated with a family history of autism as compared to a family of ADHD, and that hypo-responsivity in infancy may be an indicator of later autism and ADHD outcomes. However, better measurement of each domain is required to draw strong conclusions as many hypo-responsivity items overlapped with autistic and ADHD traits. Alterations in E/I balance may contribute to early differences in sensory responsivity but further research is required to determine the directionality of effects.

## Introduction

Although atypical sensory responsivity is traditionally associated with autism [1–3] (and included in the diagnostic criteria), recent research has suggested sensory responsivity may also be altered in other neurodevelopmental conditions, such as Attention-Deficit Hyperactivity Disorder (ADHD) [4], which commonly co-occurs with autism [5]. Despite the wide-ranging negative consequences of atypical sensory responsivity in childhood [6–8], and it being a priority area for individuals with lived experience [9], little is known about how sensory responsivity may develop differently in children with autism and/or ADHD, or the neurobiological factors that may contribute to these individual differences.

Numerous studies have reported that alterations in responsivity to sensory inputs, namely hyper-responsivity (increased response) and hypo-responsivity (decreased response), are highly prevalent in autistic individuals [3, 10, 11] and are present from early in development [12, 13]. Though the evidence base is emergent, some studies report alterations in sensory responsivity are also associated with ADHD [4, 14–16]. Older theoretical frameworks suggest that certain attributes of ADHD (e.g., hyperactive behaviour) are driven by hypo-sensitivity to sensory inputs [17], although there is limited empirical research as to the specific nature of sensory processing differences associated with ADHD. Better understanding of the nature of sensory differences associated with autism and ADHD is key to delineating shared vs. distinct markers and highlighting potentially causal mechanisms. A small number of studies have compared the sensory profiles associated with autism and ADHD directly; currently we are lacking a robust evidence base regarding the similarities and differences in sensory processing and responsivity between the two conditions. In one large clinical child and adolescent cohort, cluster-based analyses found considerable overlap in profiles associated with either an autism or ADHD diagnosis, although when scores on individual domains were inspected, autistic individuals showed greater sensory differences than people with ADHD on most subscales [18]. In an online sample, authors report evidence for a general “N” (neurodiversity) factor which loads across a range of domains, including differences in sensory sensitivities [19]. These provisional results suggest alterations in sensory processing may represent a transdiagnostic feature of neurodevelopmental conditions (i.e., a shared neuroendophenotype [5]), although they may be more prevalent or impactful in autism. However, most studies are conducted with individuals after they receive their diagnosis. To detect causal pathways, and to understand what is distinct or common between autism and ADHD in terms of pattens of sensory processing and response, studies are required that examine sensory differences before symptom emergence, and test the effect of both conditions within the same statistical model. Infant-sibling designs, where infants with a family history of neurodevelopmental conditions are recruited around birth, report infants with a family history of autism (FH-autism) who receive a diagnosis themselves have atypical sensory hyper and hypo-responsivity from early in development [13, 20–22]. One study found 3 year old children with a family history of ADHD (FH-ADHD) also score higher on parent-reported hyper and hypo-responsivity [23], but analyses did not account for the co-occurrence between hyper- and hypo-responsivity, or variation in autism FH or autistic traits in the child, limiting conclusions about specificity of effects. To our knowledge, no study has compared FH-autism and FH-ADHD siblings in terms of developmental patterns of sensory responsivity, or examined how these pattern predict emerging traits of these conditions.

In addition to delineating early developmental profiles of sensory responsivity associated with autism and ADHD, better understanding of relevant neurobiological processes that underpin variation in sensory responsivity is required to inform evidence-based intervention. One proposed mechanism that underpins differences in sensory processing and responsivity is the relative ratio of excitatory to inhibitory activity in the brain (E/I balance), with inhibition in particular thought to govern the processing of sensory inputs through mechanisms such as neuronal tuning [24]. There is some evidence that alterations in markers of E/I balance are correlated with atypical sensory responsivity in autistic children and adults [7, 25–28]. There is also evidence for the role of inhibition in the processing of sensory information; in vivo GABA levels in the sensorimotor cortex are associated with thresholds for tactile discrimination [29], drugs that increase GABAergic inhibition in the brain increase perceptual suppression during binocular rivalry [30], and differences in visual processing between autistic and neurotypical participants are abolished when autistic people are administered a GABA receptor agonist [31]. However, nearly all studies are conducted in older individuals, limiting inference about directionality of effects. Focusing on the early infant period is especially pertinent to E/I balance as animal models suggest this is a critical period for dynamic increases in inhibitory activity in the somatosensory cortex [32], and when GABA shifts from a depolarizing (i.e., excitatory) to a hyperpolarizing (i.e., inhibitory) function [33], the timing of which sets the stage for development of circuits relevant to maintaining E/I balance throughout the lifespan [34]. One infant study, in an overlapping cohort to the current analytic sample, reported that although 10-month old FH-autism infants showed differences in repetition suppression to tactile stimulation (a marker of GABAergic inhibition), no association was found between tactile suppression index and parent-rated tactile sensory seeking [35]. This may be in part due to difficulties capturing observable individual differences in tactile sensory seeking at such a young age.

Recently, novel methods have been developed that allow extraction of proxy metrics of global brain E/I balance from non-invasive electroencephalography (EEG) recordings (see [36] for a methodological review): the aperiodic 1/f signal [37, 38]. The exponent of this slope captures individual differences in the relative amplitude of aperiodic high frequency to low frequency oscillations, and is associated with the ratio of excitatory to inhibitory neural activity in animal and simulation studies [38]. Recent findings from longitudinal infant cohorts report that higher aperiodic exponents precede and predict autistic traits in childhood [39, 40] yet how this putative marker of E/I balance relates to sensory responsivity has not yet been explored.

There is evidence from infant-sibling studies that certain brain and cognitive features are more clearly associated with autism outcomes when measured earlier in development [41, 42]. This may reflect the emerging influence of other modifying domains that come online later in life that obscure brain-behaviour associations (i.e., developmental moderators) or signal early cortical adaptation. The idea of developmental specificity is of particular relevance to E/I balance, as it has been proposed that the brain may attempt to adapt to alterations to E/I balance early in the lifespan, which in turn could lead to group effects reversing later in development (as the brain ‘overcorrects’ itself) [43]. Indeed, family history of ADHD is associated with higher exponents in infancy (aged 1 month), but a diagnosis of ADHD in older children (aged 11 – 17 years) is associated with lower exponents [44]. Similar biological adaptation processes are noted elsewhere, for example arterial remodelling in response to atherosclerotic plaques, [45] neural reorganization such that non-speech areas predict language outcomes in children with cochlear implants [46], and increased levels of obesity in adults exposed to famine in utero [47]. To understand developmentally specific effects, associations between E/I balance and sensory responsivity need to be tested at multiple points in the early infant period.

To address limitations of current knowledge, we estimate trajectories of hyper and hypo-responsivity, and test whether these are predicted by family history, and predictive of childhood neurodevelopmental traits. We estimate developmental change as previous work finds that change rates in sensory responsivity are the most robust predictors of autistic traits [12]. To understand the specificity of effects with regard to FH, we include terms that index the presence or absence of FH-autism and FH-ADHD as separate predictors in the same statistical model, which allows one to test the effect of one form of FH over and above the other. We predict that infants with FH-autism would show increased hyper- and hypo-responsivity, and increases in both would be associated with higher autistic traits at 36 months. We predict that infants with FH-ADHD would show increased hypo-responsivity only, which would in turn predict ADHD traits at 36 months, in line with theoretical frameworks [17]. We examine associations between E/I balance (indexed by the aperiodic exponent) and hyper- and hypo-responsivity, as listed in our pre-registered analysis (https://osf.io/sm4f8). To explore the idea that there may be specific periods where E/I balance is the critical determinant of sensory processing, we examined associations at 5, 10, and 14 months of age.

## Methods

Infants were recruited as part of a longitudinal prospective study (Studying Autism and ADHD in the early years; STAARS) (see [48]) for more details. Participants were recruited between 2013–2019 from a volunteer database, community flyers, internet adverts and clinical networks. Informed written consent was provided by the parent(s). The study was approved by the National Research Ethics Service and the Research Ethics Committees of Birkbeck, University of London and King’s College London. All experiments and assessments were performed in accordance with relevant guidelines and regulations.

161 infants completed visits at approximately 5, 10, 14, 24 and 36 months of age. Infants were assigned group membership based on information on clinical diagnoses and scores on various screening measures (see Supplement Materials, section SM1 and Table S1). For sample description – the final sample consisted of the following groups: infants in the FH-autism group had at least one first-degree relative with a community clinical diagnosis of autism (n = 80), infants in the FH-ADHD group had at least one first-degree relative with a community clinical diagnosis or probable research diagnosis of ADHD (n = 31), and infants in the FH-typical group had at least one older sibling with typical development and no known autism or ADHD in first-degree family members (n = 29). Infants with a family history of both autism and ADHD formed the FH-autism + ADHD group (n = 21). Although the sample was recruited on the basis of these four groups to ensure sufficient variability of the different forms of FH, all infants were coded 1 or 0 for FH-autism (present/absent; n = 96/55) and 1 or 0 for FH-ADHD (present/absent; n = 49/102) and these two variables were entered simultaneously into regression models to examine the unique effects of one type of family history over and above the effects of the other. 12 infants from the sample received a research diagnosis of autism at 36 months (see Supplement Materials section SM1 for more detail on how this was reached). The current sample includes infants who had at least one measurement of sensory responsivity between 10 and 36 months (n = 151).

### Sensory responsivity

Sensory hyper-responsivity and hypo-responsivity were measured at 10, 14, 24 and 36 months with the 48-item Infant Toddler Sensory Profile (ITSP) [49]. A composite low threshold score was calculated by combining the sensory sensitivity and sensation avoiding subscales [50]. We used the total score from the low threshold quadrant as an index of hyper-responsivity (25 items) and scores from the low registration quadrant as an index of hypo-responsivity (11 items) (as the description of behaviours captured by the low registration quadrant are conceptually comparable to the construct of hypo or decreased responsivity [51]). Scores were reverse scored to aid interpretability.

### Neurodevelopmental traits

Autistic traits were measured at 36 months using the Preschool form of the Social Responsiveness Scale – 2 [52]. The SRS consists of 65 items and is designed to measure autistic traits in the general population. ADHD traits were measured at 36 months using the Preschool Child Behavior Checklist DSM Attention Deficit/Hyperactivity Problems subscale [53]. which comprises six items that measure inattentive and hyperactive behaviours. Traits were measured in all children.

### EEG acquisition and procedure

EEG was collected at 5, 10 and 14 months. EEG was recorded using an EGI (Philips Neuro, Oregon, USA) 128-electrode Hydrocel Sensor Net, online referenced to Cz. EEG was collected at 500 Hz at 5 and 10 months and 1000 Hz at 14 months. Infants were seated on their caregiver’s lap, 60 cm from a screen, in a sound attenuated and electrically-shielded room. Stimuli consisted of naturalistic social (women singing) or non-social (toys moving) videos designed to produce calm attention. Videos were 1 min in length and presented up to 3 times, interspersed between other tasks as part of a longer session. EEG was bandpass filtered (0.1–100 Hz), and 1 s segmented. Data was manually cleaned in NetStation 4.5; segments with excessive artefact, where infants were not looking at the video, or with >25 noisy channels were excluded. Infants with <10 artefact-free trials in either condition were excluded (as in [54]). Noisy channels were interpolated from neighbouring channels using spline interpolation. 1 s non-overlapping segments were referenced to the average reference, imported into Matlab, detrended and subjected to a Fast Fourier Transform (FFT). Power values were calculated and averaged across artefact-free segments in 1 Hz bins from all electrodes excluding those on the outer rim of the net (electrodes 17, 43, 48, 49, 56, 63, 68, 73, 81, 88, 94, 99, 107, 113, 119, 120, 125, 126, 127, 128).

### Extraction of E/I metrics from EEG

The fitting oscillations and one over f (FOOOF) algorithm was used to obtain individual aperiodic exponent values [37]. When the power spectrum is plotted on a log–log axis (i.e., power on the y axis, frequency on the x axis), the aperiodic exponent is equivalent to the coefficient of the regression line. A steeper slope, and thus higher exponent, is thought to be indicative of greater inhibition [38]. We parameterized spectra in the 1–20 Hz range, and only exponents from model fits with R^2^ ≥ 0.95 and > 20 trials were retained (see eFig. 1 and eTable 2 in Supplement 1 for a breakdown and analysis of EEG attrition). Other settings were: peak width limits = 2, 8, maximum number of peaks = 4, peak threshold = 0.1, aperiodic mode = ‘fixed’. Previous work finds no differences in aperiodic exponent for the social as compared to non-social videos [40]; therefore, aperiodic exponents were averaged for the social and non-social videos (as outlined in our pre-registered analysis, although we tested condition differences post-hoc to check this could not explain the pattern of results, see Supplementary Materials SM2). To minimise the impact of outliers, values were winsorized such that the 5% of the lowest/highest values were recoded to the value of the 5th/95th percentile.

### Statistical analysis

All analyses were conducted in Stata v16. Analyses were pre-registered (https://osf.io/sm4f8; see Supplementary Materials SM3 for deviations). Normality of ITSP data was confirmed before running growth curve models. Given that many scales that purport to measure hypo-reactivity often include items that overlap strongly with attention and social communication skills (which could impact on interpretation of results), we first estimate models for hyper-responsivity in isolation before estimating joint models that also include hypo-responsivity in the next step (as in [55]). First, a univariate latent growth curve mediation model was estimated, specifying correlated latent factors of intercept (baseline, here 10 months) and slope (change over time) for sensory hyper-responsivity (see Figure S2; Model 1a). Paths were specified from two binary family history variables (FH-autism absent /present and FH-ADHD absent/present, each coded a 0/1) and sex (male/female, coded as 0/1) to sensory responsivity intercepts, slopes and neurodevelopmental traits, and from sensory responsivity intercepts and slopes to neurodevelopmental traits. As the binary FH variables were entered simultaneously in all models, the FH-autism coefficient indicates the effect of FH-autism, holding all other variables in the model constant (and the same interpretation is true for the FH-ADHD and sex coefficients). In these models, the FH-autism*FH-ADHD interaction was added in a second step to aid interpretation of main effects and test the presence of non-additive effects between FH-autism and FH-ADHD. Time-varying age effects were accounted for by specifying age at visit as a predictor of observed ITSP scores at each wave. Second, a joint model was estimated, which also included the intercept and slope of hypo-responsivity (Model 1b). Next, we then ran these two-step models with the 5-, 10- and 14-month aperiodic exponents and number of available EEG trials as a predictor of intercept and slope (one model for each EEG timepoint; Models 2a and 2b). FH-autism, FH-ADHD, FH-autism*FH-ADHD and sex were included as covariates. To aid model convergence, 36-month timepoints were excluded from these models as we were interested in the associations between aperiodic exponents and sensory responsivity, rather than neurodevelopmental traits. To check differences in general cognitive development was not driving the pattern of effects, we re-ran all models looking at prediction of outcomes including Mullen Scales of Early Learning composite score as a time-varying covariate. To check any significant effects of aperiodic exponent were not driven by general cognitive development, we re-ran models including concurrent Early Learning composite score as an additional predictor in the 5, 10 and 14-month models (i.e., measured at the same age as EEG was collected). The pattern of results remained the same in all models, apart from one change in significance level noted below. All models were estimated with maximum likelihood to account for missing data. We report both unstandardised (b) and standardized (B) coefficients. The final Stata code can be accessed at 10.17605/OSF.IO/C6MQ4.

## Results

### Associations between autism/ADHD family history, sensory responsivity, and childhood traits

See Table 1 and Table S3 for descriptive statistics.

**Table 1:** Characteristics of Analytic Sample.

Mean (SD) unless otherwise indicated	Total sample (N = 151)	Typical FH (n = 26)	FH-autism (n = 76)	FH-ADHD (n = 29)	FH-autism + ADHD (n = 20)	
Sex n (%)						
Male	83 (55%)	15 (58%)	38 (50%)	18 (62%)	12 (60%)	
Female	68 (45%)	11 (42%)	38 (50%)	11 (38%)	8 (40%)	
Ethnicity n (%)						
White	121 (83%)	23 (92%)	54 (74%)	27 (93%)	17 (89%)	
Mixed Race	15 (10%)	1 (4%)	10 (14%)	2 (7%)	2 (11%)	
Asian	7 (5%)	1 (4%)	6 (8%)	0	0	
Black	2 (1%)	0	2 (3%)	0	0	
Other	1 (1%)	0	1 (1%)	0	0	
Parent education n (%)						
Primary/Secondary	41 (28%)	2 (8%)	17 (23%)	10 (34%)	12 (63%)	
BA/BSc or above	105 (72%)	23 (92%)	56 (77%)	19 (66%)	7 (37%)	
Child Assessments						
5 m (n = 97)	Age at visit	5.32 (0.57)	5.39 (0.50)	5.35 (0.66)	5.15 (0.38)	5.25 (0.45)
	Mullen ELC	84.48 (10.04)	85.30 (8.71)	83.02 (10.93)	85.54 (9.01)	87.75 (9.72)
10 m (n = 144)	Age at visit	10.06 (0.62)	10.00 (0.63)	10.03 (0.52)	10.20 (0.91)	10.11 (0.46)
	Mullen ELC	87.57 (14.90)	89.23 (12.30)	88.49 (15.00)	84.76 (15.86)	85.42 (16.83)
14 m (n = 136)	Age at visit	14.29 (0.65)	14.23 (0.61)	14.31 (0.64)	14.22 (0.80)	14.37 (0.60)
	Mullen ELC	77.82 (12.25)	79.50 (11.76)	78.28 (12.00)	79.08 (11.12)	72.53 (14.50)
24 m (n = 123)	Age at visit	24.75 (1.33)	24.61 (1.16)	24.91 (1.56)	24.68 (1.09)	24.38 (0.72)
	Mullen ELC	104.05 (20.63)	115.30 (15.53)	100.71 (20.92)	106.86 (21.21)	96.94 (17.12)
36 m (n = 118)	Age at visit	37.18 (1.81)	36.83 (1.82)	37.21 (1.46)	37.35 (2.69)	37.19 (1.52)
	Mullen ELC	113.25 (19.52)	129.94 (11.41)	108.10 (18.53)	118.39 (18.83)	105.93 (19.90)
	SRS total	39.95 (31.75)	23.37 (9.46)	42.91 (32.39)	35.55 (24.19)	61.00 (48.52)
	CBCL ADHD total	4.30 (3.28)	3.05 (2.16)	4.22 (3.28)	4.77 (3.49)	5.79 (3.93)

*CBCL* child behavior checklist, *ELC* early learning composite, *FH* family history, *SRS* social responsiveness scale.

In Model 1a (hyper-responsivity only, full results in Table 2), FH-autism was positively associated with the slope of hyper-responsivity (*b* = 1.94, 95% CI [0.15, 3.72], *p* = 0.033, *B* = 0.26). Both the intercept and the slope of hyper-responsivity were positively associated with autistic traits (intercept: *b* = 0.035, 95% CI [0.03, 0.05], *p* < 0.001, *B* = 0.59 ; slope: *b* = 0.08, 95% CI [0.05, 0.11], *p* < 0.001, *B* = 0.43) and ADHD traits at age 3 years (intercept: *b* = 0.18, 95% CI [0.134, 0.23], *p* < 0.001, *B* = 0.63; slope: *b* = 0.33, 95% CI [0.16, 0.50], *p* < 0.001, *B* = 0.36). No sex effects were observed (all *ps* > 0.34).

**Table 2:** Associations between Family History of Autism and ADHD and Trajectories of Sensory Hyper-Responsivity (Model 1a).

	b	95% CI		*p*	*B*
**Hyper-responsivity intercept**					
FH-Autism	1.385	−2.992	5.762	0.535	0.059
FH-ADHD	1.895	−2.583	6.372	0.407	0.079
FH-Autism*FH-ADHD	3.036	−5.945	12.018	0.508	0.091
Sex	−0.920	−4.910	3.070	0.651	−0.041
**Hyper-responsivity slope**					
FH-Autism	1.936	0.153	3.720	0.033	0.256
FH-ADHD	1.197	−0.636	3.029	0.201	0.154
FH-Autism*FH-ADHD	−1.055	−4.730	2.619	0.574	−0.098
Sex	−0.503	−2.138	1.132	0.546	−0.069
**36** **m SRS Total**					
Hyper-responsivity intercept	0.035	0.026	0.045	0.000	0.587
Hyper-responsivity slope	0.081	0.047	0.114	0.000	0.434
FH-Autism	0.158	−0.048	0.365	0.133	0.113
FH-ADHD	0.065	−0.140	0.270	0.533	0.045
FH-Autism*FH-ADHD	−0.004	−0.408	0.400	0.985	−0.002
Sex	−0.038	−0.216	0.140	0.676	−0.028
**36** **m CBCL ADHD Total**					
Hyper-responsivity intercept	0.183	0.134	0.231	0.000	0.603
Hyper-responsivity slope	0.332	0.164	0.500	0.000	0.355
FH-Autism	0.212	−0.827	1.252	0.689	0.030
FH-ADHD	0.598	−0.444	1.640	0.261	0.082
FH-Autism*FH-ADHD	−0.586	−2.633	1.462	0.575	−0.058
Sex	−0.444	−1.347	0.460	0.336	−0.065

FH-Autism*FH-ADHD terms were added in a separate step so FH-Autism and FH-ADHD terms should be interpreted as direct effects.

*CBCL* child behavior checklist, *FH* family history, *SRS* social responsiveness scale.

**Fig. 1:**
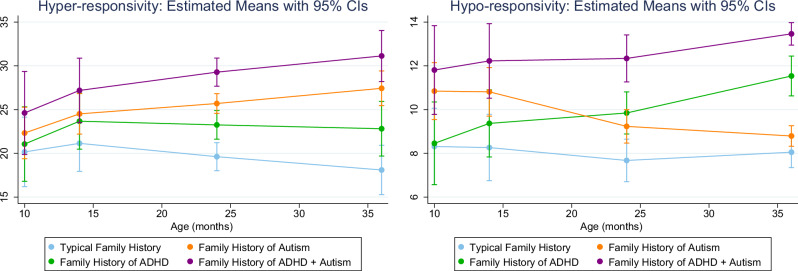
FH-autism was significantly associated with a steeper slope of hyper-responsivity between 10–36 months whereas FH-ADHD was significantly associated with a steeper slope of hypo-responsivity.

**Fig. 2:**
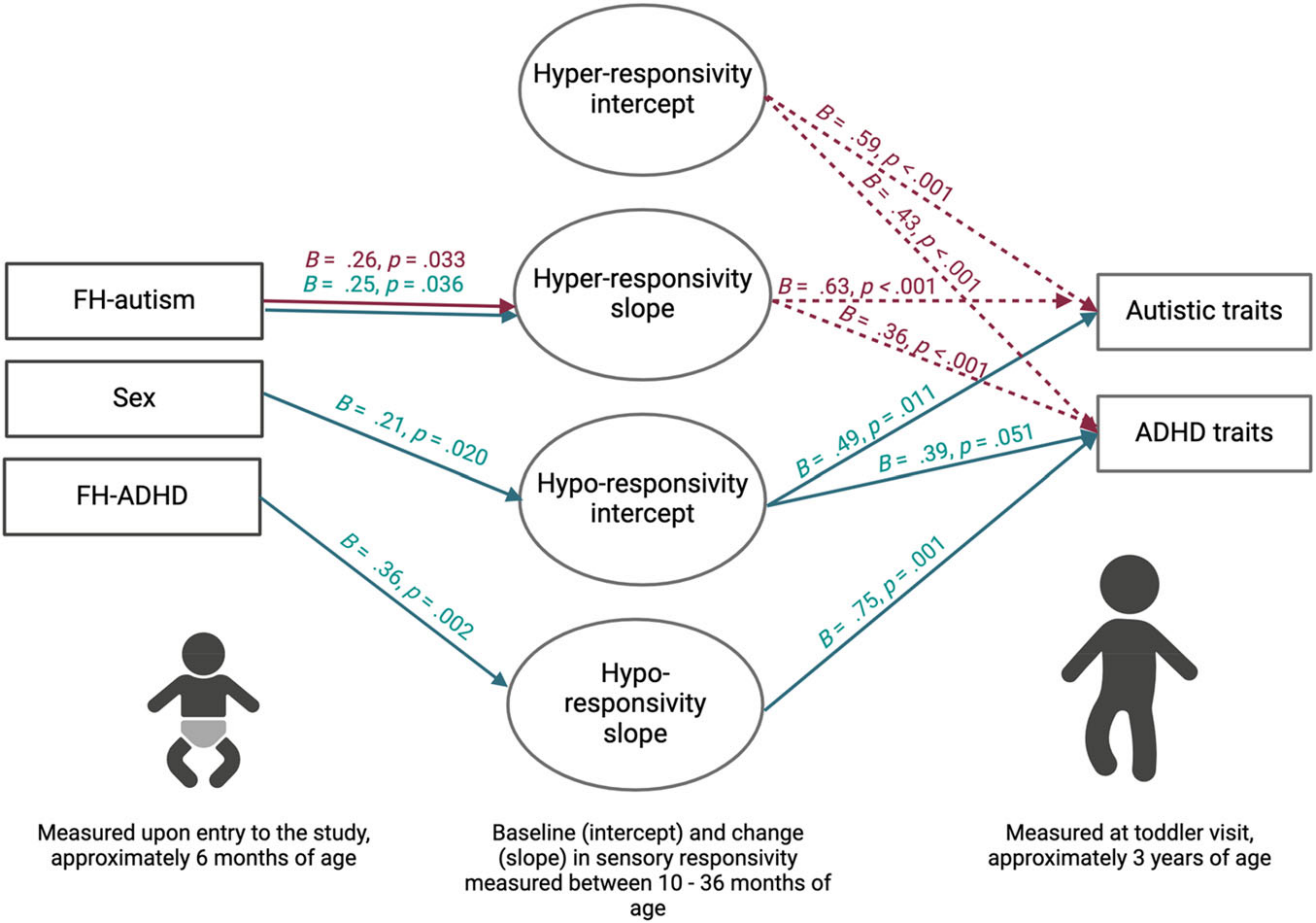
Associations between Autism and ADHD Family History Status, Sex, Trajectories of Sensory Responsivity and Toddler Neurodevelopmental Traits. Red dashed pathways indicate effects only significant in Model 1 (hyper-responsivity only), red pathways indicate effects significant in Model 1 and Model 2 (hyper- and hypo-responsivity). Green paths indicate effects in significant Model 2. The FH-autism*FH-ADHD interaction was entered in a second step and non-significant in all tests and so not included here. B = standardised estimates. Created with BioRender.com.

In Model 1b (hyper and hypo-responsivity, full results in Table S4), FH-autism remained positively associated with the slope of hyper-responsivity (*b* = 1.90, 95% CI [0.12, 3.67], *p* =0.036, *B* = 0.25). FH-ADHD was positively associated with the slope of hypo-responsivity (*b* = 1.41, 95% CI [0.51, 2.30], *p* = 0.002, *B* = 0.36). Males had higher scores than females for the hypo-responsivity intercept (*b* = −2.27, 95% CI [−4.18, −0.35], *p* = 0.020, *B* = −0.21). Hyper-responsivity was significantly associated with hypo-responsivity (intercept covariance: *B* = 0.85, *p* < 0.001, slope covariance: *B* = 0.71, *p* < 0.001). Neither the intercept or slope of hyper-responsivity was significantly associated with later autistic or ADHD traits (*ps* > 0.10). A higher hypo-responsivity intercept was associated with higher autistic traits (*b* = 0.06, 95% CI [0.01, 0.11], *p* = 0.011, *B* = 0.49) and ADHD traits at age 3 (*b* = 0.25, 95% CI [0.00, 0.49], *p* = 0.051, *B* = 0.39), although the latter on the boundary of significance. There was a positive association between the slope for hypo-responsivity and ADHD traits at aged 3 (*b* = 1.40, 95% CI [0.58, 2.22], *p* = 0.001, *B* = 0.75). When Mullen Early Learning composite was included as an additional time-varying covariate, the association between the hypo-responsivity intercept and ADHD traits at aged 3 was no longer statistically significant (*p* = 0.078). See Fig. 1 for estimates of group trajectories in sensory responsivity and Fig. 2 for an overall summary of the pattern of results.

### Associations between EEG markers of E/I balance and sensory responsivity

In Model 2a (full results in Table 3), 5-month and 10-month aperiodic exponent were positively associated with the intercept of sensory hyper-responsivity (5 months: *b* = 22.93, 95% CI [2.20, 43.67], *p* = 0.030, *B* = 0.27; 10 months: *b* = 17.58, 95% CI [0.91, 34.25], *p* = 0.039, *B* = 0.20). 14-month exponent was not associated with hyper-responsivity intercept or slope (ps > 0.80).

**Table 3:** Associations between Global Metrics of Excitation/Inhibition Balance and Trajectories of Sensory Hyper-Responsivity (Model 2a).

	b		95% CI	*p*	*B*
**Hyper-responsivity intercept**					
5 m aperiodic exponent	22.934	2.202	43.666	0.030	0.267
5 m number of trials	0.046	−0.030	0.122	0.237	0.141
10 m aperiodic exponent	17.581	0.911	34.252	0.039	0.203
10 m number of trials	−0.030	−0.091	0.030	0.327	−0.098
14 m aperiodic exponent	0.066	−18.995	19.127	0.995	0.001
14 m number of trials	−0.032	−0.093	0.029	0.298	−0.126
**Hyper-responsivity slope**					
5 m aperiodic exponent	0.288	−8.447	9.022	0.949	0.010
5 m number of trials	0.020	−0.011	0.052	0.205	0.188
10 m aperiodic exponent	−0.209	−7.407	6.989	0.955	−0.007
10 m number of trials	0.002	−0.024	0.029	0.864	0.023
14 m aperiodic exponent	−0.850	−8.296	6.596	0.823	−0.032
14 m number of trials	0.006	−0.017	0.030	0.597	0.075

Although effects for FH-autism, FH-ADHD, FH-autism*FH-ADHD and sex were included as before, they are omitted from the table for brevity. Models for the 5-, 10- and 14-month data were run separately. *FH* family history.

**Fig. 3:**
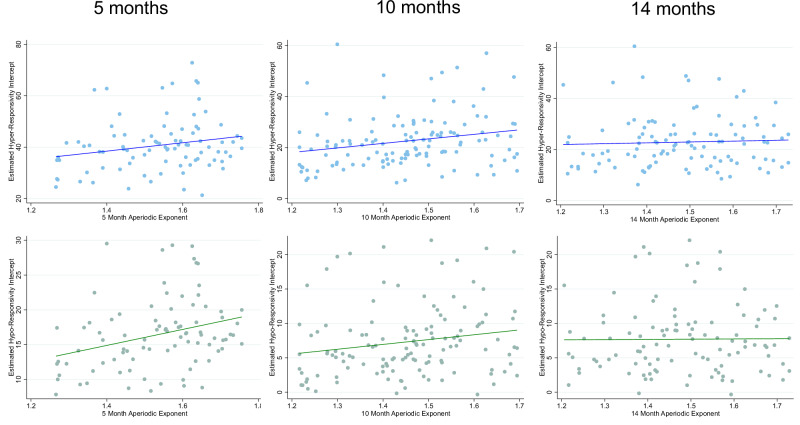
Associations between Metrics of Global E/I Balance (Aperiodic Exponent) and Hyper- (top blue panels) and Hypo-Responsivity (bottom green panels). Associations were significant with Hyper-responsivity at 5 and 10 months only.

In Model 2b (full results in Table S5), 5-month and 10-month aperiodic exponent remained positively associated with the intercept of sensory hyper-responsivity (p = 0.039 and =0.035 respectively). The association between 5-month aperiodic exponent and hypo-responsivity intercept was at significance (*b* = 10.59, 95% CI [−0.16, 21.35], *p* = 0.054, *B* = 0.25). 14-month aperiodic exponent was not associated with sensory responsivity. No associations were observed with number of retained EEG trials, aside from an at-significance association between number of trials at the 14-month assessment and the slope of hypo-responsivity (*b* = 0.01, 95% CI [0.00, 0.02], *p* = 0.051, *B* = 0.26). See Fig. 3 for a summary of results.

## Discussion

Disturbances of sensory responsivity are commonly associated with autism, and emerging evidence suggests potentially also with ADHD. However, critical questions remain as to how early these differences emerge in development, the different type of sensory profile associated with autism as compared to ADHD, and the neurobiological mechanisms that may drive variation in sensory responsivity. We present analyses of a prospective longitudinal cohort of infants, followed from 5 – 36 months, with repeated measurement of sensory responsivity. By running statistical models that include terms for both FH-autism and FH-ADHD in the same model, we tested the effect of one form of FH whilst adjusting for the impact of the other. Results showed that *only* a family history of autism (FH-autism) was associated with steeper increases in hyper-responsivity. Conversely, *only* a family history of ADHD (FH-ADHD) was associated with steeper increases in hypo-responsivity. A higher intercept (equivalent to estimated level at 10-month baseline) and steeper slope of hyper-responsivity was associated with *both* higher later autistic and ADHD traits at age 3 years in bivariate models, however in jointly estimated models which also included developmental trajectories of hypo-responsivity these associations became non-significant. Here, a higher intercept of hypo-responsivity was associated with being male, and having *both* higher later autistic and ADHD traits at age 3 years; a steeper slope of hypo-responsivity was also associated with *only* higher ADHD traits. We did not find any evidence for interactive effects between FH-autism and FH-ADHD as interaction terms were non-significant. Alterations in markers of global E/I balance, potentially indicative of enhanced inhibition, were associated with increased hyper-responsivity (and there was evidence to suggest increased hypo-responsivity also).

In line with our predictions, FH-autism infants displayed a steeper slope (greater increase between 10 – 36 months) in hyper-responsivity over the first three years of life. Conversely, and in line with theoretical frameworks (e.g., [17]), family history of ADHD was associated with steeper slope of hypo-responsivity. Our results suggest a form of double dissociation when using statistical approaches that can account for the co-occurrence of FH-autism and FH-ADHD, in that each type of genetic liability showed associations with a different form of sensory responsivity. These heritable differences in sensory responsivity suggest that although alterations in sensory responsivity have been associated with both autism and ADHD, the specific form these alterations take is key.

With regards to toddler outcomes, in our first set of models, we found the intercept and slope of hyper-responsivity were positively associated with both autistic and ADHD traits in toddlerhood. However, when we include hypo-responsivity in jointly estimated models, effects for hyper-responsivity became non-significant. Instead, we see the intercept of hypo-responsivity was associated with both autistic and ADHD traits, and the slope of hypo-responsivity was associated with ADHD traits only. The change in the pattern of results for hyper-responsivity could indicate two things. Either all effects are driven by hypo-responsivity and any significant findings with hyper-responsivity can be explained by their co-occurrence. Alternatively, it could be that many measures of hypo-responsivity, including that used in the present analyses, are in part capturing emerging autism and ADHD characteristics. Indeed, the ITSP hypo-responsivity subscale captures a broad range of behaviours, some of which overlap with our outcomes of interest (e.g., attention, regulation, emerging autism behaviours). By including hypo-responsivity in all models, we may have inadvertently overshadowed any associations with hyper-responsivity due to construct overlap in the hypo-responsivity variable. This is why we chose to run a model with hyper-responsivity in isolation before adding in hypo-responsivity. Previous work has largely not included hyper- and hypo-responsivity in the same analytic model (despite evidence of their co-occurrence), bringing into question the specificity of previous findings. One prospective community study that included both types of sensory responsivity in the same model reported both hyper- and hypo-responsivity were associated with later autism outcomes [12], although this work covered a wider developmental window (6 months – 7 years). Another study, using a subset of the aforementioned sample, found certain developmental profiles of sensory responsivity were associated with both autism and ADHD outcomes [14]. A further potential explanation of discrepancies between past and current results is that in the current study we focused on autistic traits in toddlerhood, whereas most other studies focused on diagnosis. However, there is evidence that subclinical autistic and ADHD traits share genetic aetiology with the extreme end of scores [56–58], suggesting the pattern of behavioural and cognitive correlates should be comparable. Future studies should investigate if a similar pattern of effects is found when diagnosis is used as the outcome variable of interest.

Measurement issues aside, the association between autistic traits and hypo-responsivity intercept (essentially indexing the level of hypo-responsivity at 10 months) is consistent with reports that hypo-responsivity is more prevalent in autism in early childhood [10], and predicts later autism symptoms in infant-siblings [59]. Early hypo-responsivity may have cascading effects on cognitive development as it could lead to important cues being missed during critical periods; hypo-responsivity in infancy is associated with lower levels of joint attention and language [60], decreased neural response to social stimuli and fewer social approach behaviours [61]. The fact that the intercept of hypo-responsivity was also associated with ADHD traits raises the suggestion that this sensory response pattern may not be a specific marker for later autistic characteristics, but also relevant for emerging ADHD behaviours (and thus may function as a shared neuroendophenotype).

The association between the slope of hypo-responsivity and ADHD traits at age 3 extends findings from meta-analyses that report broad alterations in sensory processing in the early years are predictive of ADHD outcomes [4]. The lack of association with hyper-responsivity goes against previous reports [23, 62], although many of these studies did not account for the co-occurrence of ADHD and autism, were conducted in older samples, or did not include hyper- and hypo-responsivity in the same analytic model. One interpretation of our results is that hypo-responsivity is a heritable characteristic which drives the emergence of ADHD behaviours – ADHD traits may represent a form of compensation for early hypo-responsivity, such that the child learns to sample the environment more rapidly and randomly to overcome for a decreased response to external inputs. However, an alternative explanation is that the current link between parent-reported hypo-responsivity and ADHD traits could be due to a misinterpretation of differences in attention control that are characteristic of ADHD as an apparent lack of responsivity as cues are ‘missed’. The previously mentioned measurement issues should also be held in mind, as measurement overlap between hypo-responsivity subscale and our outcome domains could explain the pattern of results.

Objective measures of sensory processing and response, along with other potential confounders, are required to test evidence for these different explanations. If the role of hyper- or hypo-responsivity is supported through other studies with more precise measurement, it would highlight the importance of sensory responsivity differences in children with a family history of autism and/or ADHD, which could be useful information for both parents and clinicians when trying to understand unusual patterns of sensory behaviour early in development. For example, a child that seems to avoid or disengage from playgroups/nursery may be doing this to avoid being overstimulated by sensory input rather than not being interested in social interaction. This increased awareness could facilitate more sensitive patterns of caregiving for children with sensory hyper- and hypo-responsivity, and be used to guide screening and intervention efforts (e.g., providing more stimulating sensory activities for children with a family history of ADHD, or considering sensory triggers and overwhelm in children with a family history of autism). Finally, we found males were rated as more hypo-responsive than females (as in general population cohorts [12]), emphasising the need to account for sex in analytic models.

In terms of putative neurobiological mechanisms, our pre-registered hypothesis on the role of E/I balance was supported. Increased aperiodic exponent values, indicative of enhanced inhibitory activity, were associated with greater hyper-responsivity. However, our pre-registered plan had specified analysis of EEG data at 14 months, whereas we found significant effects at 5- and 10-months only. Thus, given the potential for false positives with multiple statistical tests, current results (including those pertaining to FH status) require replication. Results concur with other infant-sibling studies that find brain or cognitive features are more strongly predictive of later autism outcomes when measured earlier in development [41, 42]. Crucially, results demonstrate the role of alterations in E/I balance early in the lifespan, potentially before initial effects become confounded by developmental compensation [43] or later emerging moderating factors [63]. Although, as discussed above, the links between hyper-responsivity and later autistic characteristics requires further study, the association between hyper-responsivity and our putative marker of E/I balance suggests that alterations in E/I balance could be one early step on the neurobiological pathway to autism outcomes. Alternatively, even if we take it at face value that there is no direct path from early hyper-responsivity to autism outcomes, this does not preclude interactive effects with later emerging modifier characteristics. Theoretical models of neurodevelopment (e.g., AMEND; [63]) posit heritable early perturbations in sensori-motor processing may be necessary but not sufficient causal factors to engender neurodivergent outcomes – these frameworks emphasise it is the interaction between these early differences and later modifiers that will predict the emergence of neurodevelopmental conditions. In support of this, previous work finds aperiodic exponents at 10 months are positively associated with autistic traits in early childhood, but only in those with relatively lower executive capacities [40]. Finally, we note the association between 5-month aperiodic exponent and hypo-responsivity was at significance, and the standardized coefficients of effect were comparable for hyper and hypo-responsivity (*B* = 0.26 and 0.25), suggesting enhanced inhibition in early development may alter sensory processing at a system-wide level [24]. Possible mechanisms of effect include disruption in evoked gamma oscillatory activity, which in turn alters the temporal sharpening of cortical sensory responses [64]. To move to a more precise understanding of how E/I balance shapes sensory responsivity in humans early in the lifespan, more translational work is needed to bridge the gap between the global EEG markers used presently, and more specific levels of neural function, including single cell recordings, neurotransmitter release and local circuit activity. Much work on the mechanisms of E/I balance has been conducted in animals, and in the case of human research, most studies finding increased excitation in autistic people come from adult populations. This could in part reflect homeostatic compensation for early increases in inhibition during critical periods. Examining these processes early in development is key to determining primary of effects.

One limitation is the reliance on parent ratings for behavioural phenotypes, which could be impacted by rater biases (e.g., sex effects could in part reflect gendered expectations of child behaviour) and imprecise definitions (e.g., some items on the ITSP could be seen to relate to emerging autistic characteristics rather than sensory responsivity). Future studies should ideally combine precisely defined parent and observational measures of sensory responsivity, and better explore the impact of other developmental factors on observed sensory response (e.g., executive function and regulatory abilities). A further limitation is the fact that parents in our sample was largely highly educated and primarily White (83%); others report that trajectories of parent-reported sensory responsivity differ by ethnicity [14], thus replicating current results in samples with greater diversity of ethnicity and socio-economic status is needed to understand the generalisability of findings. Finally, as expected, groups differed in cognitive functioning (although we note the pattern of results did not change when we included scores of general cognitive development as a covariate). Infants in the FH-autism group were also primarily defined by virtue of having an older sibling with a confirmed diagnosis, whereas the FH-ADHD group were more often defined by parents scoring above threshold on ADHD screener instruments. The latter was due to ADHD being diagnosed much later in childhood [65], making it harder to recruit on the basis of sibling diagnostic status. Thus, although both groups were defined on the basis of a first degree relative, it is unknown whether having a parent vs. a sibling with autism/ADHD has a differential impact on key characteristics of the target infant. Inclusion of genomic information (e.g., polygenetic scores for autism and ADHD outcomes) could help to disentangle shared vs. distinct effects as these can be measured in the same way within the same individual.

To summarize, results showed early increases in hyper-responsivity in infants with a family history of autism, whereas a family history of ADHD was associated with increasing hypo-responsivity. Results also suggest increased hypo-responsivity may function as a general marker for later traits of neurodivergence as it was associated with traits of both autism and ADHD later in childhood, although the field requires more precise measures of sensory processing and responsivity to draw stronger conclusions. Early alterations in E/I balance may contribute to differences in childhood sensory responsivity, highlighting a putative neurobiological mechanism that could inform assessment and intervention. Future research should explore how differences in sensory processing may arise from alterations in E/I balance, and in turn how these translate to observable differences in responsivity.

## Supplementary information

Supplemental Material

## Data Availability

The datasets analysed during the current study are subject to the BASIS/STAARS data sharing policy (see www.basis-network.org). Statistical analysis scripts can be found at OSF 10.17605/OSF.IO/C6MQ4.
