# Supplementary material for: Cortical markers of excitation/inhibition balance are associated with sensory responsivity from infancy in longitudinal cohorts enriched for autism and ADHD
Source: Transl Psychiatry. 2025 Dec 18;15:532. doi: 10.1038/s41398-025-03791-9 (PMC12722238; doi:10.1038/s41398-025-03791-9)
Supplement: Supplementary file 1 — Supplemental Material [file 41398_2025_3791_MOESM1_ESM.docx]

**Supplementary Materials**

**SM1. Sample recruitment and assessment of family history status**

Participants were recruited for a longitudinal study running from 2013 to 2019. All infants were born full-term (gestational age >36 weeks). At the time of enrolment, none of the infants had a known medical or developmental condition. Infants could be enrolled in the study if they either had a first degree relative with autism spectrum disorder, a first degree relative with diagnosed or probable ADHD, or no first-degree relatives with either diagnosis. Information about diagnostic status was ascertained through a number of methods. Before families enrolled in the study, a telephone screening form was used to determine the presence of autism and ADHD in family members. During their infant’s visit to the lab, the parent/caregiver also completed a “Medical and Psychiatric History Interview” with the researcher. The telephone screening form and this formal interview at a study visit were the primary sources of information about diagnostic status for either the parent or sibling. Typically, childhood diagnoses of ADHD were reported for older siblings, whilst parents were diagnosed with ADHD either in childhood or adulthood. In addition, we asked for medical updates at each study visit and re-administered the Medical and Psychiatric History Interview at the 2-year timepoint. We also requested diagnostic letters (pertaining to either the older sibling’s or the parent’s diagnosis) and asked parents to complete the DAWBA ^1^ ASD and ADHD sections and these were reviewed by the senior clinician (TC). In addition, parents completed the Conners ^2-4^ (for ADHD) and the Social Communication Questionnaire ^5^ and Social Responsiveness Scale ^6^ for autism on the family member (either the older sibling or parent) with a diagnosis and where possible all other family members. This information is used to characterise our sample rather than for exclusionary purposes since, in the UK, NHS clinical diagnoses follow a gold-standard procedure including collation of information from parents, teachers and from in-person assessment that is beyond the scope of this study and more accurate than simple questionnaire measures.

A proportion of children/parents had suspected ADHD, but this had not yet been confirmed by clinical services (see Table S1). This is expected since we were targeting children with infant siblings, and often there can be significant delays in the diagnostic process for ADHD. Further, up to 30% of autistic children meet criteria for ADHD when prospectively assessed ^7^. Given the nature of the co-occurrence between autism and ADHD and our longitudinal study, sometimes family members would have a suspected diagnosis of ADHD at study entry that would be confirmed later in the study; on other occasions, a family would enrol on the basis of an autism diagnosis in an older sibling but by the end of the study, they would report that the same sibling was now undergoing assessment for suspected additional ADHD.

For those who reported suspected ADHD, screening questionnaires were used to examine the probable existence of ADHD. Inclusion decisions were reviewed by the project management team.

Specifically, for siblings (6 years or older) we used a shortened adapted version of the Conners 3 ^2^. Current behaviours that parents reported as occurring either “often” or “frequently” were scored. All included children met a minimum threshold for inclusion of i) 6 ADHD symptoms on either the hyperactivity/impulsivity scale (consisting of item numbers: 3, 43, 45[54]*, 61, 69[99]*, 71, 93, 98, 104) or the inattention scale (consisting of item numbers: 2, 28, 35, 47, 68[79]*, 84, 95, 97, 101), and ii) a positive score on the impairment scale (at least 2 out of 3 impairment items, consisting of item numbers: 106, 107, 108). Note that * indicates that these two items were collapsed into a single question in the adapted screening form.

For siblings aged less than 6 years, we used a shortened adapted version of the Conners Early Childhood ^4^. Behaviours that parents reported as occurring either “often” or “frequently” were scored. All included children met a minimum threshold for inclusion of i) 9 ADHD symptoms on the inattention/hyperactivity scale (consisting of item numbers: B8, B12, B22, B34, B42, B47, B49, B55, B65, B72, B74), and ii) a positive score on the impairment scale (at least 2 out of 3 impairment items, consisting of item numbers: IM1, IM2, IM3).

For parents, a shortened adapted version of the Conners Adults ADHD Rating Scale ^3^, either self or observer report. Current behaviours that parents reported as occurring either “often” or “frequently” were scored. All included parents met a minimum threshold for inclusion of 5 ADHD symptoms on either the hyperactivity/impulsivity scale (consisting of item numbers: 2, 4, 6, 8, 16, 18, 22, 25, 27) or the inattention scale (consisting of item numbers: 1, 9, 13, 14, 19, 21, 26, 29, 30). Of note, the adult version of the Conners does not include impairment questions.

Families who screened positive on this instrument were then included as a confirmed case (see Table S1). However, it remains likely that within families with ASD, rates of actual ADHD are higher than those captured by our 1/0 diagnostically-based rating system.

For the target infant-sibling, a best estimate clinical diagnosis of autism was reached at 36 months based on DSM-5 criteria. This was informed by, but not dependent on, scores on the ADOS-2, ADI-R, SCQ, Vineland, and Mullen/WASI, researcher observations on the visit, and additional parent-reported information, by experienced researchers with research reliability on the ADOS-2 and overseen by a senior licensed clinical psychologist.

**SM2. Additional Condition Analyses of EEG Data**

We specified in our pre-registered analyses that we would average across social and non-social conditions as we did not have any specific predictions about condition effects, or whether associations between the aperiodic exponent and sensory responsivity would differ between social vs. non-social stimuli. However, at reviewers request we checked for condition differences in the aperiodic exponent at each time point (using mixed effect models with age at assessment, sex, FH-autism, FH-ADHD, condition, number of trials for social condition and number of trials for non-social condition as predictors). These tests relate to the difference in mean level of aperiodic exponent between conditions, rather than evidence for a condition-by-aperiodic exponent interaction. The results for the condition term are as follows: 5m: condition coef = -.03, 95% CIs [-.06, .01], p=.068, 10m: condition coef = .02, 95% CIs [.01, .03], p = .042, 14m: condition coef = .03, 95% CIs [.01, .05], p = .002.

**SM3. Deviations from Pre-Registered Analytic Plan**

We deviated from our pre-registered analyses in the following ways: we include neurodevelopmental traits as outcomes in our mediation models, and model testing EEG correlates of sensory responsivity use the 5- and 10-month EEG datapoints in addition to the 14-month data to allow us to explore evidence for developmental compensation. We included an interaction between autism FH and ADHD FH in addition to main effects to account for any non-linear effects, and the 10-month timepoint of ITSP measurement to aid estimation of developmental trajectories as models would not converge with the 14 to 36-month timepoints only. Finally, to aid convergence, the error variance of observed ITSP variables was constrained to be the same over time within domain. Although we also pre-registered additional analyses using measurement of sensory responsivity from subscales of temperament questionnaires (the perceptual sensitivity scale on the Infant Behavioural Questionnaire; IBQ, and Early Child Behaviour Questionnaire; ECBQ; ^8,9^), on-going investigations in our group have highlighted low within-construct correlations over time (e.g., ^10^), and we found low contemporaneous correlations between ITSP hyper-responsivity and IBQ/EBCQ scores between 10 and 24 months (*rs* = .08 - .37). As such, we were less confident these measures were capturing stable differences in sensory responsivity and thus did not analyse them further. Finally, due to COVID-related delays in data acquisition, we plan to conduct the pre-registered analyses using data from the LEAP cohort and childhood BASIS timepoints in a future manuscript.

**Table S1. Categorisation of the elevated likelihood cohorts**

|  | **FH-autism** | **FH-ADHD** | **FH-autism+ADHD** |
| --- | --- | --- | --- |
| Parent reported diagnosis in older sibling | 64 | 7 | 15 |
| Parent reported diagnosis in parent | 3 | 13 | 2 |
| Parent reported diagnosis in both older sibling+parent | 2 | 1 | 1 |
| Screened parent (for ADHD traits) |  | 2 | 1 |
| Screened older sibling (for ADHD traits) |  | 1 | 0 |

| T**imepoint** | **FH-autism** | **FH-ADHD** | **Sex** |
| --- | --- | --- | --- |
| 5 Month EEG (n=77) | χ2 =0.10, *p* = .75 | χ2 = 3.01, *p* = .08 | χ2 = 1.18, *p* = .28 |
| 10 Month EEG (n=123) | χ2 = 0.12, *p* = .73 | χ2= 0.17, *p* = .68 | χ2 = 3.76, *p* = .05 |
| 14 Month EEG (n=103) | χ2 = 0.84, *p* = .36 | χ2 = 0.03, *p* = .87 | χ2 = 0.32, *p* = .57 |

**Table S2. Analysis of Family History and Sex as Predictors of EEG Data Missingness**

**Table S3. Summary statistics for key variables in growth curve models, split by group**

|  | Typical FH (n=26) | | FH-autism (n=76) | | FH-ADHD (n=29) | | FH-autism + ADHD (n=20) | |
| --- | --- | --- | --- | --- | --- | --- | --- | --- |
|  | Mean | SD | Mean | SD | Mean | SD | Mean | SD |
| 5m aperiodic exponent | 1.53 | 0.13 | 1.55 | 0.13 | 1.51 | .16 | 1.49 | .12 |
| 5m number of trials | 99.27 | 32.91 | 103.08 | 34.14 | 105.99 | 36.82 | 106.70 | 37.95 |
| 10m ITSP hyper-responsivity | 20.02 | 8.40 | 22.25 | 15.29 | 20.35 | 7.46 | 22.43 | 13.93 |
| 10m ITSP hypo-responsivity | 9.83 | 4.83 | 11.02 | 7.19 | 7.87 | 3.72 | 10.76 | 7.09 |
| 10 m aperiodic exponent | 1.45 | .14 | 1.46 | .13 | 1.45 | .11 | 1.42 | .14 |
| 10m number of trials | 89.64 | 44.62 | 97.75 | 35.92 | 100.68 | 27.63 | 89.08 | 38.57 |
| 14m ITSP hyper-responsivity | 21.18 | 7.99 | 23.59 | 14.82 | 22.44 | 9.82 | 26.72 | 13.23 |
| 14m ITSP hypo-responsivity | 8.13 | 4.54 | 10.65 | 6.96 | 10.50 | 6.05 | 12.26 | 6.70 |
| 14m aperiodic exponent | 1.55 | .13 | 1.47 | .13 | 1.46 | .14 | 1.46 | .17 |
| 14m number of trials | 111.17 | 44.63 | 117.37 | 40.01 | 126.17 | 43.05 | 117.41 | 35.11 |
| 24m ITSP hyper-responsivity | 18.36 | 8.63 | 23.93 | 14.83 | 25.58 | 14.33 | 27.33 | 18.57 |
| 24m ITSP hypo-responsivity | 7.05 | 3.15 | 8.47 | 7.12 | 10.82 | 6.86 | 11.53 | 8.40 |
| 36m ITSP hyper-responsivity | 15.86 | 7.34 | 26.46 | 16.99 | 19.22 | 11.63 | 30.14 | 25.78 |
| 36m ITSP hypo-responsivity | 6.93 | 3.88 | 8.64 | 6.76 | 10.12 | 5.54 | 12.64 | 11.98 |

FH = family history; ITSP = Infant Toddler Sensory Profile

**Table S4. Associations between Family History of Autism and ADHD and Trajectories of Sensory Hyper- and Hypo-Responsivity (Model 1b)**

|  | b | 95% CI | | *p* | *B* |
| --- | --- | --- | --- | --- | --- |
| **Hyper-responsivity intercept** | |  |  |  |  |
| FH-Autism | 1.353 | -3.069 | 5.774 | 0.549 | 0.057 |
| FH-ADHD | 1.802 | -2.722 | 6.326 | 0.435 | 0.074 |
| FH-Autism*FH-ADHD | 3.020 | -6.057 | 12.097 | 0.514 | 0.090 |
| Sex | -1.005 | -5.038 | 3.028 | 0.625 | -0.044 |
| **Hyper-responsivity slope** | |  |  |  |  |
| FH-Autism | 1.899 | 0.122 | 3.675 | 0.036 | 0.250 |
| FH-ADHD | 1.205 | -0.622 | 3.033 | 0.196 | 0.154 |
| FH-Autism*FH-ADHD | -1.096 | -4.756 | 2.564 | 0.557 | -0.101 |
| Sex | -0.518 | -2.147 | 1.111 | 0.533 | -0.070 |
| **Hypo-responsivity intercept** | |  |  |  |  |
| FH-Autism | 1.909 | -0.188 | 4.006 | 0.074 | 0.168 |
| FH-ADHD | 0.273 | -1.872 | 2.418 | 0.803 | 0.023 |
| FH-Autism*FH-ADHD | 1.193 | -3.110 | 5.496 | 0.587 | 0.074 |
| Sex | -2.265 | -4.177 | -0.354 | 0.020 | -0.206 |
| **Hypo-responsivity slope** | |  |  |  |  |
| FH-Autism | -0.319 | -1.185 | 0.548 | 0.471 | -0.083 |
| FH-ADHD | 1.405 | 0.512 | 2.298 | 0.002 | 0.357 |
| FH-Autism*FH-ADHD | -0.177 | -1.961 | 1.608 | 0.846 | -0.033 |
| Sex | 0.270 | -0.524 | 1.064 | 0.505 | 0.073 |
| **36m SRS Total** |  |  |  |  |  |
| Hyper-responsivity intercept | 0.011 | -0.010 | 0.033 | 0.298 | 0.196 |
| Hyper-responsivity slope | 0.036 | -0.025 | 0.096 | 0.246 | 0.196 |
| Hypo-responsivity intercept | 0.060 | 0.014 | 0.106 | 0.011 | 0.490 |
| Hypo-responsivity slope | 0.097 | -0.035 | 0.229 | 0.149 | 0.268 |
| FH-Autism | 0.190 | -0.048 | 0.429 | 0.118 | 0.137 |
| FH-ADHD | 0.001 | -0.219 | 0.221 | 0.994 | 0.001 |
| FH-Autism*FH-ADHD | -0.034 | -0.416 | 0.348 | 0.862 | -0.017 |
| Sex | 0.013 | -0.171 | 0.196 | 0.892 | 0.010 |
| **36m CBCL ADHD Total** |  |  |  |  |  |
| Hyper-responsivity intercept | 0.085 | -0.030 | 0.200 | 0.147 | 0.284 |
| Hyper-responsivity slope | -0.179 | -0.532 | 0.174 | 0.321 | -0.191 |
| Hypo-responsivity intercept | 0.246 | -0.001 | 0.492 | 0.051 | 0.392 |
| Hypo-responsivity slope | 1.398 | 0.578 | 2.217 | 0.001 | 0.750 |
| FH-Autism | 1.309 | -0.084 | 2.701 | 0.065 | 0.184 |
| FH-ADHD | -0.616 | -1.895 | 0.663 | 0.345 | -0.084 |
| FH-Autism*FH-ADHD | -0.805 | -2.950 | 1.340 | 0.462 | -0.080 |
| Sex | -0.676 | -1.758 | 0.406 | 0.221 | -0.098 |

Note: FH-Autism*FH-ADHD terms were added in a separate step so FH-Autism and FH-ADHD terms should be interpreted as direct effects. CBCL = Child Behavior Checklist, FH = family history, SRS = Social Responsiveness Scale

**Table S5. Associations between Global Metrics of Excitation/Inhibition Balance and Trajectories of Sensory Hyper- and Hypo-Responsivity (Model 2b)**

|  | **b** | **95% CI** | | ***p*** | ***B*** |
| --- | --- | --- | --- | --- | --- |
| **Hyper-responsivity intercept** | | | | | |
| 5m aperiodic exponent | 22.173 | 1.119 | 43.228 | 0.039 | 0.256 |
| 5m number of trials | 0.047 | -0.029 | 0.123 | 0.222 | 0.144 |
| 10m aperiodic exponent | 18.058 | 1.285 | 34.830 | 0.035 | 0.206 |
| 10m number of trials | -0.033 | -0.094 | 0.028 | 0.286 | -0.106 |
| 14m aperiodic exponent | -0.647 | -19.797 | 18.504 | 0.947 | -0.008 |
| 14m number of trials | -0.037 | -0.097 | 0.024 | 0.236 | -0.141 |
| **Hyper-responsivity slope** | | | | | |
| 5m aperiodic exponent | 0.083 | -8.936 | 9.102 | 0.986 | 0.003 |
| 5m number of trials | 0.020 | -0.012 | 0.051 | 0.232 | 0.181 |
| 10m aperiodic exponent | -0.669 | -7.819 | 6.481 | 0.854 | -0.023 |
| 10m number of trials | 0.003 | -0.023 | 0.030 | 0.819 | 0.030 |
| 14m aperiodic exponent | -1.345 | -8.776 | 6.086 | 0.723 | -0.051 |
| 14m number of trials | 0.003 | -0.021 | 0.026 | 0.816 | 0.033 |
| **Hypo-responsivity intercept** | | | | | |
| 5m aperiodic exponent | 10.411 | -0.610 | 21.432 | 0.064 | 0.249 |
| 5m number of trials | 0.006 | -0.033 | 0.045 | 0.769 | 0.037 |
| 10m aperiodic exponent | 6.472 | -1.633 | 14.578 | 0.118 | 0.153 |
| 10m number of trials | -0.019 | -0.049 | 0.011 | 0.209 | -0.125 |
| 14m aperiodic exponent | 0.212 | -8.609 | 9.032 | 0.962 | 0.005 |
| 14m number of trials | -0.018 | -0.046 | 0.010 | 0.196 | -0.147 |
| **Hypo-responsivity slope** | | | | | |
| 5m aperiodic exponent | -1.532 | -5.913 | 2.850 | 0.493 | -0.106 |
| 5m number of trials | 0.004 | -0.012 | 0.020 | 0.609 | 0.074 |
| 10m aperiodic exponent | -0.318 | -3.737 | 3.101 | 0.855 | -0.022 |
| 10m number of trials | 0.001 | -0.011 | 0.014 | 0.841 | 0.025 |
| 14m aperiodic exponent | 0.994 | -2.512 | 4.501 | 0.578 | 0.075 |
| 14m number of trials | 0.011 | 0.000 | 0.022 | 0.051 | 0.257 |

Note: Although effects for FH-autism, FH-ADHD, FH-autism*FH-ADHD and sex were included as before, they are omitted from the table for brevity. Models for the 5-, 10- and 14-month data were run separately. FH = family history

10 months (n=144)

EEG data collected (n=139)

Excluded if R2 <.95 (n=129 retained for non-social videos, n=133 retained for social videos)

Excluded if < 20 trials (n=125 retained for non-social videos, n=124 retained for social videos)

Non-social and social video data averaged, data available from n=128

5 months (n=97)

EEG data collected (n=96)

Excluded if R2 <.95 (n=83 retained for non-social videos, n=84 retained for social videos)

Excluded if < 20 trials (n=82 retained for non-social videos, n=80 retained for social videos)

Non-social and social video data averaged, data available from n=88

14 months (n=136)

EEG data collected (n=109)

Excluded if R2 <.95 (n=100 retained for non-social videos, n=105 retained for social videos)

Excluded if < 20 trials (n=94 retained for non-social videos, n=99 retained for social videos)

Non-social and social video data averaged, data available from n=105

Figure S1. Summary of EEG Data Retention

Also had at least one measurement of sensory responsivity n=77

(79% of infants who attended 5 month visit)

Also had at least one measurement of sensory responsivity n=123

(85% of infants who attended 10 month visit)

Also had at least one measurement of sensory responsivity n=103

(76% of infants who attended 14 month visit)

FH-autism, FH-ADHD, FH-autism*FH-ADHD, sex

Age at assessment

ITSP Low Threshold 14m

ITSP Low Threshold 10m

ITSP Low Threshold 24m

ITSP Low Threshold 36m

Age at assessment

Age at assessment

Age at assessment

Figure S2. Latent Growth Curve Model

Dashed boxes indicate specified loadings between latent growth factors and observed data. FH = family history; ITSP = Infant and Toddler Sensory Profile

1v

1v

1.75

2.75

0.5

1v

1v

36m autistic traits

36m ADHD traits

**References**

1. Goodman R, Ford T, Richards H, Gatward R, Meltzer H. The Development and Well-Being Assessment: Description and Initial Validation of an Integrated Assessment of Child and Adolescent Psychopathology. *Journal of Child Psychology and Psychiatry*. 2000;41(5):645-655. doi:<https://doi.org/10.1111/j.1469-7610.2000.tb02345.x>

2. Conners CK. *Conners 3rd Edition*. Multi-Health Systems Assessments; 2008.

3. Conners CK, Erhardt D, Sparrow E. *Conners Adults ADHD Rating Scale*. Multi-Health Systems Assessments; 1999.

4. Conners CK. *Conners Early Childhood* Multi-Health Systems Assessments; 2009.

5. Rutter M, Bailey A, Lord C. *Social Communication Questionnaire (SCQ)*. Western Psychological Services,; 2003.

6. N. CJ, C. G. *Social responsiveness scale (SRS)*. Western Psychological Services; 2012.

7. Simonoff E, Pickles A, Charman T, Chandler S, Loucas T, Baird G. Psychiatric disorders in children with autism spectrum disorders: prevalence, comorbidity, and associated factors in a population-derived sample. *Journal of the American Academy of Child & Adolescent Psychiatry*. 2008;47(8):921-929.

8. Gartstein MA, Rothbart MK. Studying infant temperament via the Revised Infant Behavior Questionnaire. *Infant Behavior and Development*. 2003/02/01/ 2003;26(1):64-86. doi:<https://doi.org/10.1016/S0163-6383(02)00169-8>

9. Putnam SP, Gartstein MA, Rothbart MK. Measurement of fine-grained aspects of toddler temperament: The Early Childhood Behavior Questionnaire. *Infant Behavior and Development*. 2006/07/01/ 2006;29(3):386-401. doi:<https://doi.org/10.1016/j.infbeh.2006.01.004>

10. Narvekar N, Carter Leno V, Pasco G, et al. The roles of sensory hyper and hyposensitivity in infancy in understanding later anxiety and emerging autistic traits. *PsyArXiv*. 2022;doi:10.31234/osf.io/yqjsu
